# Comparison of gut microbial communities, free amino acids or fatty acids contents in the muscle of wild *Aristichthys nobilis* from Xinlicheng reservoir and Chagan lake

**DOI:** 10.1186/s12866-022-02440-1

**Published:** 2022-01-20

**Authors:** Yuting Lu, Peijun Zhang, Wei Li, Jia Liu, Xinchi Shang, Yi Cheng, Yuehong Li

**Affiliations:** 1 College of Animal Science and Technology, Jilin Agricultural University, Changchun, 130118 China; 2 Ministry of education laboratory of animal production and quality security, Jilin Agricultural University, Changchun, 130118 China; 3 Health monitoring and Inspection Center of Jilin Province, Changchun, 130062 China; 4 Department of Clinical Laboratory, China-Japan Union Hospital, Changchun, 130021 Jilin Province China

**Keywords:** Bighead carp (*Aristichthys nobilis*), Gut microbial community, Free amino acids, Fatty acids

## Abstract

**Background:**

Fish is favored by consumers, while amino acids and fatty acids are the main nutrients of muscle. At present, it has been found that the gut microbial community may be involved in the regulation of host material anabolism. Juvenile and adult bighead carp (*A. nobilis*) from Chagan lake and Xinlicheng reservoir were selected, and divided into four groups to compare the differences of gut microbial communities, free amino acid and fatty acids in muscle.

**Results:**

The results showed that fish in different lakes or ages contained specific microbiota, the gut microbial structure was similar, but the microbial content was significantly different. Gut microbial abundance of juvenile fish in Chagan lake was significantly higher than that of other groups. Phylum level analysis *Proteobacteria* was the dominant gut bacteria of fish in both adult and juvenile fish from two separate lakes. *Actinobacteria* was another dominant bacterial phylum in juvenile fish in both lakes. Contents of free amino acids and fatty acids in muscle were detected, and the relationships between them and gut microbial communities were analyzed. Bighead carp grew from juvenile to adult, *Actinobacteria* abundance decreased (*P* < 0.05) and *Proteobacteria* increased (*P* < 0.05). *Proteobacteria* was positively correlated with the contents of Thr, Lys, Pro, Asp, Gly and Glu, *Actinobacteria* was negatively correlated with Met and His. Meanwhile, EPA and DHA were positively correlated with *Proteobacteria,* EPA and DHA were not significantly associated with *Actinobacteria*.

**Conclusion:**

It was speculated that the contents of free amino acids and fatty acids in muscle might be affected by the difference of gut microbiota, thus affecting the taste and nutritional quality.

**Supplementary Information:**

The online version contains supplementary material available at 10.1186/s12866-022-02440-1.

## Introduction

Bighead carp (*Aristichthys nobilis*), together with black carp (*Mylopharyngodon piceus*), grass carp(*Ctenopharyngodon idellus*) and silver carp (*Hypophthalmichthys molitrix*) is the main economically important freshwater fish in China [1]. It is a typical filter-feeding fish and has the elegant name of ‘water scavenger’. In China, the demand for bighead carp (*A. nobilis*) has increased due to its nutritional value, distinctive flavor, high protein, low fat, low cholesterol and overall quality. The nutritional value and organoleptic characteristics of fish meat are influenced by variety, size, and degree of sexual maturity [2, 3]. The quality of fish meat is also affected by a complex set of characteristics factors such as texture, the amount of water, ions, amino acids, fatty acids [4].

The gut tract of an animal is a complex ecosystem with a huge microbial community. Gut microbial communities are symbiotic with the host and play important roles in host physiology, nutrition and health [5–8]. Gut microbial communities convert food into various nutrients for the host body, and the host provides a suitable environment and food energy to microbial communities. Normally, a dynamic balance among the various classes of gut microbial communities, but the balance is broken that gut microbial community is also related to the occurrence and progress of obesity [9], inflammatory bowel diseases (IBD), etc. [10]. Pengfei Xu et al. [11] found that melatonin prevents obesity through modulation of gut microbiota in mice. In addition, gut microbial communities also regulated substance metabolism, tissue and organ development [12], and immune system maturation [13, 14] by changing host genes expression genes. Consequently, researchers came up with the concept that gut microorganisms served as an “organ”. For fish, gut microbial communities not only helps digest food but facilitate mucosal immunity [15], host-derived probiotics *Enterococcus casseliflavus* improve resistance against *Streptococcus iniae* infection in *Oncorhynchus mykiss* via immunomodulation [16].

Fish are important for consumption as food resources, one of the reasons people favor fish is that it tastes delicious [17]. The taste of fish depends on many factors, including the contents of free amino acids [18] and fatty acids [19]. Almost all amino acids elicit taste, and different amino acids produce different tastes. Further to that, fatty acids are essential nutrients for the body, especially the brain. The polyunsaturated fatty acids DHA and EPA can affect memory and mood [20, 21]. Previous studies showed that the difference in intestinal microbiota of Chinese mitten crabs from different habitats might be related to the variation in the distinctive flavor of Chinese mitten crabs [22]. And wild fish are more popular with consumers as to taste than farmed fish, suggesting that the environment in which fish are raised may also affect the quality of the fish [4]. At present, few studies have examined the gut microbial communities of bighead carp from different habitats and no reports have shown the association of gut microbial communities with fatty acids and free amino acids in muscle. Therefore, it is interesting to explore the differences of intestinal microbes in different habitats and through data parameters to establish the correlation between intestinal microbes and fatty acids and amino acids. Chagan lake is the largest natural lake in Jilin Province in China, the water is typical saline-alkaline water, the winter fishing spectacle of Chagan lake has been included in the provincial intangible cultural heritage list of Jilin Province, and the main species fished is bighead carp. Xinlicheng reservoir is a comprehensive reservoir for drinking water supply, flood control and irrigation. The water is neutral. In addition to providing water for daily use, it also provides various economically important fish every year, of which bighead carp is the majority. It has the reputation as the ‘Pearl of Spring City’ because of its beautiful environment and abundant resources. Microorganisms are the most diverse and plentiful group of organisms on earth, and the microbial community is very sensitive to the changes in the environment. Some studies use the sensitivity and stability of microbial communities to monitor water quality [23, 24]. Wu et al. found that the salinity had a great influence on the microbial community structure, and the diversity decreased with the increase of salinity in their experiments on the treatment of synthetic saline wastewater in sequencing batch reactors [25]. Similarly, the study of groundwater microbial diversity and the community composition in the salt-fresh water transition zone was found seawater had the lowest taxon richness and evenness [26]. These were the direct influences of the external environment on the microbial community, while the gut is a small and complex microbial ecosystem with its self-regulation mechanism. Kevin et al. found that temperature significantly impacted the community structure and membership of the tadpole gut, specifically, tadpoles in the warm treatment exhibited higher abundance of the phylum *Planctomycetes* and the genus *Mycobacterium* [27]. This was the indirect influences of the external environment on the microbial community of gut. While, saline and alkali is an important index affecting aquatic organisms. The effects of saline and alkali on the structure and diversity of fish gut microorganism have not been reported, and the effects of gut microorganism structure and diversity on fish quality were also rarely reported.

The subjects of this experiment were wild juvenile and adult bighead carp from Chagan lake and Xinlicheng reservoir, the purpose of this research was to (i) investigate the comparative differences in the gut microbial community structures of bighead carp as well as differences in free amino acids and fatty acids contents in muscle under water conditions in Chagan lake and Xinlicheng reservoir; (ii) provide a direction for the study of the relationship between gut microbial communities and free amino acids and fatty acids contents in muscle according to the data parameters. This study provides data support for saline-alkali aquaculture, presents new biological information support for bighead carp research, and indicates directions for improving fish quality and enhancing economic performance by changing fish flavor through gut microbial ecology.

## Results

### Water physicochemical parameters

The physicochemical parameters of the water from two lakes were shown in Table 1. According to the results, the water body of Chagan lake is saline-alkaline water and the Xinlicheng reservoir is neutral water.

**Table 1:** Water quality physicochemical parameters

Sample	Chagan lake	Xinlicheng reservoir
pH	8.39 ± 0.06	7.04 ± 0.07
DO (mg/L)	6.75 ± 0.13	7.66 ± 0.09
TP (mg/L)	0.18 ± 0.03	0.12 ± 0.01
TN (mg/L)	0.78 ± 0.03	0.63 ± 0.03
NO_3_-N (mg/L)	0.41 ± 0.01	0.38 ± 0.02
NH_4_-N (mg/L)	0.16 ± 0.02	0.14 ± 0.01
COD (mg/L)	21.33 ± 0.13	18.02 ± 0.13
COD_Mn_ (mg/L)	7.57 ± 0.32	4.76 ± 0.06
Cl^−^(mg/L)	120.3 ± 4.16	35.27 ± 2.17
SO_4_^2−^(mg/L)	62.1 ± 1.75	55.42 ± 1.23
HCO_3_^2−^(mg/L)	634.2 ± 12.35	173.15 ± 4.25
Mg^2+^(mg/L)	27.3 ± 1.64	7.47 ± 0.11
Ca^2+^(mg/L)	10.8 ± 0.58	54.45 ± 0.32
Na^+^(mg/L)	302.7 ± 5.72	24.35 ± 0.07
K^+^(mg/L)	4.9 ± 0.74	6.31 ± 0.46

Data are expressed as mean ± S.D. (*n* = 9). DO, dissolved oxygen; *TN* total nitrogen; *TP* total phosphorous; NO_3_-N nitrate; NH_4_-N ammonia; *COD* chemical oxygen demand; Anions (Cl^−^ and SO_4_^2−^) and cations (Na^+^, K^+^, Ca^2+^, and Mg^2+^)

### Contents of free amino acids in muscle

Amino acids are the smallest units of proteins, and protein is the main component of muscle. Fish are palatable due to the many free amino acids in muscle that provide taste characteristics. As shown in Table 2, 17 free amino acids were identified and quantified in all of the samples, while Glu was the predominant free amino acid, accounting for 30.85, 29.74, 30.87, and 29.57% of the total free amino acids in the CJ (Chagan lake juvenile fish group), CA (Chagan lake adult fish group), XJ (Xinlicheng reservoir juvenile fish group) and XA(Xinlicheng reservoir adult fish group) samples, respectively. Moreover, Glu content of adult fish was lower than in juveniles. However, no significant differences were observed among other free amino acids, except Asp, Gly, His, Pro, Lys, and Thr (*P* > 0.05). Meanwhile, adult fish were all higher than juveniles in free amino acids contents where there were differences (Asp, Gly, His, Pro, Lys, and Thr, *P* < 0.05). This may be caused by the ability to absorb and digest and self-synthesize amino acids being more developed in adults than in juvenile fish. According to the pooled data, total free amino acids in the muscle of adult fish (218.29 ± 2.08 g/kg) from Xinlicheng reservoir were higher than in other groups (CJ, 205.69 ± 2.48 g/kg; CA, 210.73 ± 2.11 g/kg; XJ, 215.47 ± 3.37 g/kg), and significantly higher (*P* < 0.05) when compared with that from Chagan lake. Free amino acids were higher in adult fish than in juveniles in both lakes.

**Table 2:** Contents of free amino acids (g/kg, dry basis) in muscle of bighead carp (*Aristichthys nobilis*)

Free amino acids	CJ	CA	XJ	XA
Asp	0.4 ± 0.0^a^	0.5 ± 0.0^b^	1.7 ± 0.0^c^	1.7 ± 0.0^d^
Glu	63.5 ± 0.4^a^	62.7 ± 0.1^b^	66.5 ± 0.2^c^	64.5 ± 0.4^d^
Gly	7.0 ± 0.1^a^	7.6 ± 0.4^ab^	7.4 ± 0.3^ab^	7.7 ± 0.1^b^
Ala	9.3 ± 0.3	9.7 ± 0.4	9.6 ± 0.5	9.8 ± 0.2
Arg	12.8 ± 0.6	13.0 ± 0.2	12.8 ± 0.8	13.2 ± 0.6
His	4.7 ± 0.0^a^	5.1 ± 0.0^b^	0.8 ± 0.0^c^	0.9 ± 0.0^d^
Pro	16.7 ± 0.3^a^	17.8 ± 0.0^b^	22.5 ± 0.1^c^	22.4 ± 0.3^c^
Ser	6.8 ± 0.3	6.5 ± 0.2	6.7 ± 0.1	6.6 ± 0.2
Ile	9.8 ± 0.3	9.7 ± 0.4	9.6 ± 0.3	9.7 ± 0.2
Leu	13.2 ± 0.5	13.5 ± 0.5	13.2 ± 0.3	13.7 ± 0.3
Lys	13.7 ± 0.2^a^	14.5 ± 0.6^ab^	14.5 ± 0.7^ab^	15.1 ± 0.5^b^
Met	5.0 ± 0.4	5.7 ± 0.1	5.2 ± 0.1	5.5 ± 0.3
Cys	16.8 ± 0.2	16.6 ± 0.2	16.5 ± 0.1	16.7 ± 0.2
Phe	6.9 ± 0.3	7.2 ± 0.2	7.0 ± 0.3	7.2 ± 0.2
Tyr	6.0 ± 0.2	6.1 ± 0.2	6.0 ± 0.1	6.3 ± 0.1
Thr	6.1 ± 0.2^a^	6.1 ± 0.0^a^	6.1 ± 0.1^ab^	6.4 ± 0.1^b^
Val	6.5 ± 0.6	6.5 ± 0.2	6.4 ± 0.2	6.8 ± 0.3
Total	205.7 ± 2.5^a^	210.7 ± 2.1^ab^	215.5 ± 3.4^bc^	218.3 ± 2.1^c^

Data are expressed as mean ± S.D. (n = 3). The superscript letters indicate a statistically significant difference at a *P* value of < 0.05 within each row comparison. *CJ* Chagan lake juvenile fish; *CA* Chagan lake adult fish; *XJ* Xinlicheng reservoir juvenile fish; *XA* Xinlicheng reservoir adult fish

### Content of fatty acids in muscle

The muscle fatty acid profiles are listed in Table 3. A total of 25 fatty acids were detected and identified, the major fatty acids in muscle were c16:0, c18:0, c16:1, c18:1 n-9, c20:4 n-6, EPA and DHA. Palmitic acid (c16:0) was the primary saturated fatty acid (SFA). Oleic acid (c18:1 n-9) was the most abundant monounsaturated fatty acids (MUFAs). Polyunsaturated fatty acids (PUFAs) are composed of the true essential fatty acids EPA and DHA and all others. Moreover, EPA and DHA are considered essential precursors in the synthesis of eicosanoids. Fish are good sources of EPA and DHA. EPA and DHA contents were higher in adult fish than in juvenile fish, and the contents from Xinlicheng reservoir were higher than in fish from Chagan lake. And there was no statistical difference between the adult and juvenile fish from Xinlicheng reservoir. One of the criteria for estimating the biological value of lipids is the∑n-3:∑n-6 polyunsaturated fatty acid ratio. The lipids with higher ratios are considered biologically more important, and it is desirable for this ratio to be greater than 2 [28]. For adult fish in Chagan lake, the∑n-3:∑n-6 ratio was 2.1, higher than in other groups.

**Table 3:** Fatty acids composition (g/100 g total fatty acids) in muscle of bighead carp (*Aristichthys nobilis*)

Fatty acids	CJ	CA	XJ	XA
C14:0	1.7 ± 0.1^a^	2.9 ± 0.1^b^	2.2 ± 0.0^c^	2.7 ± 0.1^b^
C15:0	0.6 ± 0.0^a^	0.6 ± 0.0^a^	0.5 ± 0.0^b^	0.5 ± 0.0^b^
C16:0	18.8 ± 0.1^a^	19.7 ± 0.1^b^	20.0 ± 0.1^c^	19.4 ± 0.0^d^
C17:0	2.3 ± 0.1^a^	1.4 ± 0.0^b^	1.2 ± 0.0^c^	1.0 ± 0.0^d^
C18:0	11.2 ± 0.0^a^	11.8 ± 0.1^b^	14.6 ± 0.1^c^	14.8 ± 0.1^c^
C20:0	0.3 ± 0.0^a^	0.3 ± 0.0^a^	0.3 ± 0.0^a^	0.2 ± 0.0^b^
C21:0	0.1 ± 0.0^ab^	0.1 ± 0.0^a^	0.1 ± 0.0^b^	0.1 ± 0.0^b^
C22:0	0.2 ± 0.0^a^	0.2 ± 0.0^a^	0.1 ± 0.0^b^	0.1 ± 0.0^b^
C23:0	0.1 ± 0.0^a^	0.0 ± 0.0^b^	0.1 ± 0.0^a^	0.0 ± 0.0^b^
C24:0	0.1 ± 0.0	0.1 ± 0.0	0.1 ± 0.0	0.1 ± 0.0
Total SFAs	35.1	36.9	39.0	38.9
C16:1	6.7 ± 0.2^a^	6.1 ± 0.1^b^	8.6 ± 0.1^c^	7.3 ± 0.0^d^
C17:1	0.9 ± 0.1^a^	0.8 ± 0.0^b^	0.4 ± 0.0^c^	0.4 ± 0.0^c^
C18:1 n-9	21.3 ± 0.0^a^	20.5 ± 0.1^b^	17.0 ± 0.1^c^	16.8 ± 0.0^d^
C20:1	1.0 ± 0.0^a^	0.3 ± 0.0^b^	0.3 ± 0.0^b^	0.3 ± 0.0^b^
C22:1 n-9	0.1 ± 0.0^a^	0.1 ± 0.0^a^	0.2 ± 0.0^b^	0.1 ± 0.0^a^
C24:1	0.1 ± 0.0^a^	0.0 ± 0.0^b^	0.1 ± 0.0^a^	0.1 ± 0.0^a^
Total MUFAs	30.0	27.9	26.6	24.9
C18:2 n-6	4.7 ± 0.0^a^	4.1 ± 0.0^b^	3.9 ± 0.0^c^	3.3 ± 0.1^d^
C18:3 n-6	0.3 ± 0.0^a^	0.3 ± 0.0^a^	0.4 ± 0.0^b^	0.3 ± 0.0^a^
C20:2	1.0 ± 0.1^a^	1.0 ± 0.0^a^	0.6 ± 0.0^b^	0.5 ± 0.0^c^
C20:3 n-3	1.2 ± 0.0^a^	0.9 ± 0.0^b^	0.3 ± 0.0^c^	0.3 ± 0.0^c^
C20:3 n-6	0.8 ± 0.^0a^	0.9 ± 0.0^b^	1 ± 0.0^c^	0.9 ± 0.0^b^
C20:4 n-6	5.4 ± 0.0^a^	5.5 ± 0.0^b^	6.0 ± 0.1^c^	8.4 ± 0.0^d^
C20:5 n-3EPA	8.0 ± 0.1^a^	8.4 ± 0.1^b^	8.8 ± 0.1^c^	8.9 ± 0.0^c^
C22:2	1.3 ± 0.0^a^	1.4 ± 0.0^b^	0.0 ± 0.0^c^	0.0 ± 0.0^c^
C22:6 n-3DHA	12.2 ± 0.2^a^	12.8 ± 0.0^b^	13.2 ± 0.2^c^	13.5 ± 0.0^c^
Total PUFAs	34.9	35.3	34.3	36.3
∑n-3	21.2	22.1	22.4	22.8
∑n-6	11.3	10.8	11.3	12.9
∑n-3:∑n-6	1.9	2.1	2.0	1.8
∑(EPA + DHA)	20.2	21.2	22.0	22.4

Data are expressed as mean ± S.D. (n = 3).The superscript letters indicate a statistically significant difference at a *P* value of < 0.05 within each row comparison. *SFA* saturated fatty acid; *MUFA* monounsaturated fatty acid; *PUFA* polyunsaturated fatty acid; *CJ* Chagan lake juvenile fish; *CA* Chagan lake adult fish; *XJ* Xinlicheng reservoir juvenile fish; *XA* Xinlicheng reservoir adult fish

### 16sDNA sequence analysis of gut bacterial community diversities

The 16S rDNA of the gut bacterial microbiota of bighead carp from two lakes was sequenced. Each sample combined the contents and mucus of guts from three individual fish. A total of 861,274 reads were obtained from all samples, and the effective data accounted for 81.44 to 87.76% in all samples. The read lengths from all of the samples were mainly between 400 and 440 bp (Table S1). These findings showed that the sequencing results were reliable. The reads were clustered into operational taxonomic units (OTUs) at 97% identity. Ace and Chao1 indices were used to reveal OTU richness, Shannon and Simpson indices were used to quantify alpha diversity (Table 4). Diversity indices showed that the CJ group communities appeared to have the highest richness as compared to other communities, while the CA group communities appeared to have the lowest richness.

**Table 4:** Number of sequences analyzed, observed diversity richness (OTUs), estimated OTU richness (ACE and Chao1), the diversity index (Shannon and Simpson) and estimated sample coverage for 16S rDNA libraries of the different samples

group	OTUs^a^	ACE^b^	Chao1	Simpson	Shannon	Coverage
CA	185	278.5716	226.4375	0.2348	1.8633	0.9997
CJ	552	554.06	554.5	0.0135	5.2832	0.9999
XA	304	318.6016	331.0	0.2586	1.9111	0.9998
XJ	333	344.209	349.2353	0.2181	2.6504	0.9999

^a^ The operational taxonomic units (OTU) were defined at the 97% similarity level

^b^ The richness estimators (ACE and Chao1), diversity indices (Shannon and Simpson) and coverage percentage (coverage) were generated with Mothur programme

*CJ* Chagan lake juvenile fish; *CA* Chagan lake adult fish; *XJ* Xinlicheng reservoir juvenile fish; *XA* Xinlicheng reservoir adult fish

### Microbial community compositions and comparisons at the phylum and genus level

**Fig.1:**
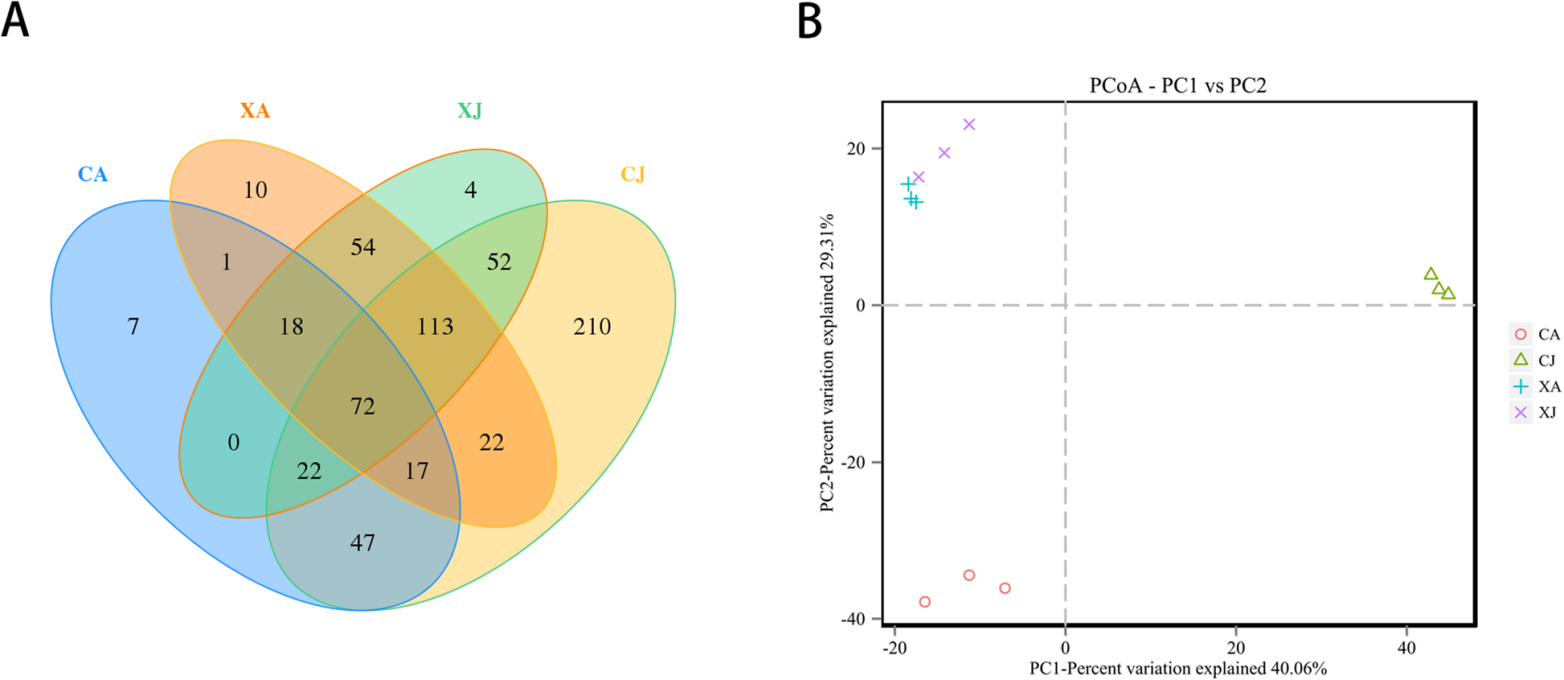
**A** Venn diagram showing the unique and shared OTUs of *A. nobilis* gut microbiome for four experimental groups. **B** Principal coordinate analysis (PCoA) of the dissimilarity between the microbial-based on unweights. CJ, juvenile fish from Chagan lake; CA, adult fish from Chagan lake; XJ, juvenile fish from Xinlicheng reservoir; XA, adult fish from Xinlicheng reservoir

According to the obtained abundance matrix of OTUs, the number of OTUs in each group and the proportions of shared and unique OTUs were intuitively shown in the Venn diagram (Fig.1 A). A total of 649 OTUs were identified. Meanwhile, there were 72 shared OTUs among four groups, and the numbers of unique OTUs of CJ, CA, XJ, XA were 210, 7, 4 and 10, respectively. To elucidate phylogenetic dissimilarity of gut microbiota, unifrac distance-based principal coordinate analysis (PCoA) was conducted and the results showed that the four groups were separated into different categories and XA group had a similar structure to that of XJ group (Fig.1 B).

**Fig. 2:**
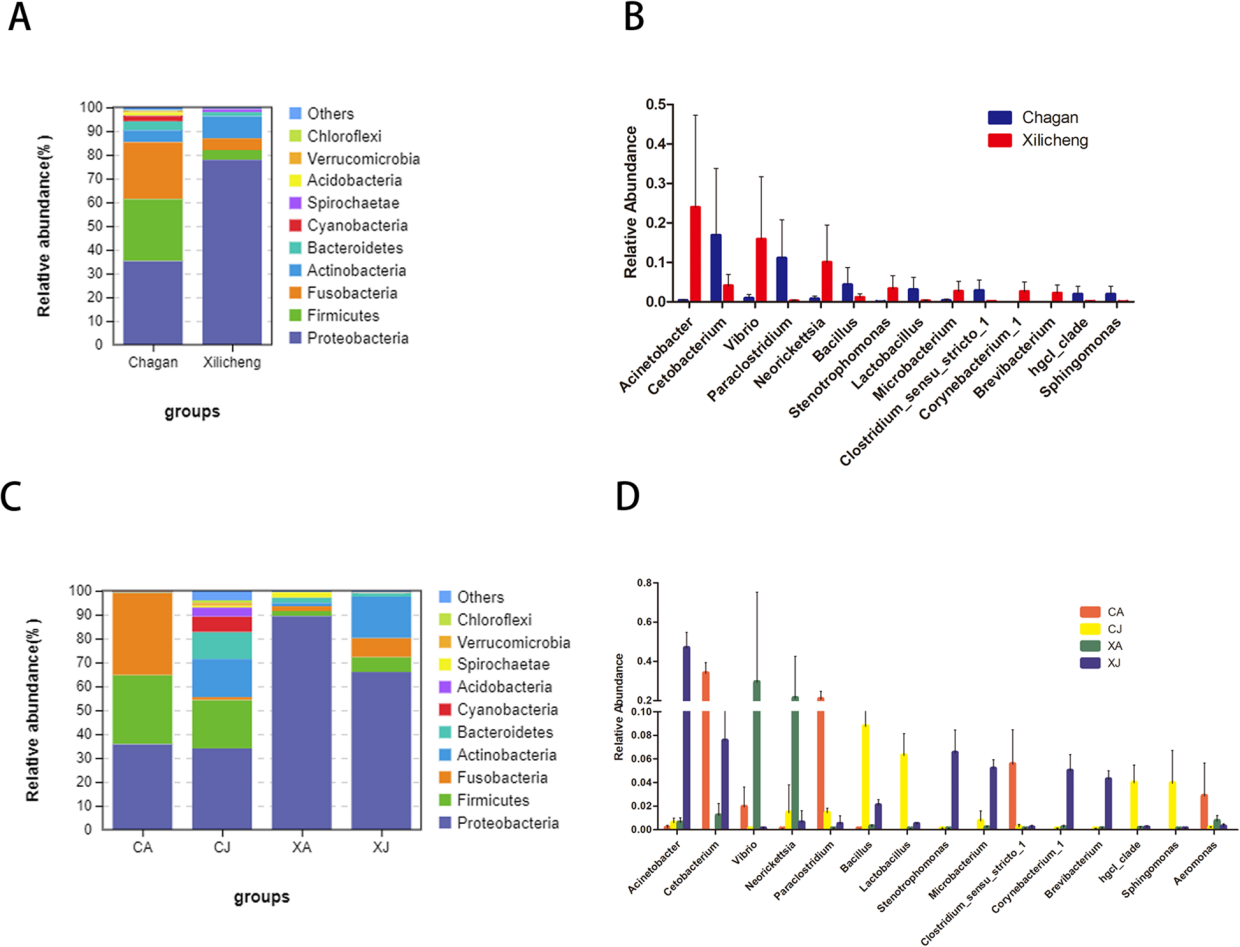
The relative abundance of gut microbial. **A**, **C** in phylum level (the top 10 dominant microbiota); **B**, **D** in genus level (abundance of microbiota > 1%). CJ, juvenile fish from Chagan lake; CA, adult fish from Chagan lake; XJ, juvenile fish from Xinlicheng reservoir; XA, adult fish from Xinlicheng reservoir

Phylum level analysis revealed that the gut bacterial communities of the bighead carp from the two lakes were mainly composed of *Proteobacteria*, *Firmicutes*, *Fusobacteria*, *Actinobacteria*, *Bacteroidetes*, and the abundance of the remaining bacterial phyla were < 3%. In the gut bacterial community of the bighead carp from Chagan lake, the abundance of *Proteobacteria* was 35.18% followed by *Firmicutes* (26.13%), *Fusobacteria* (23.97%), *Actinobacteria* (5.07%), whereas the abundance of the remaining bacterial were < 5%. Meanwhile, in Xinlicheng reservoir *Proteobacteria* was at 77.89% followed by *Actinobacteria* (9.26%), whereas the abundance of all other phyla were < 5% (Fig.2 A, Table S2). Although living in the same environment, the juvenile and adult fish dominant gut bacterial community was different and simultaneously the bacterial abundance in the intestinal tract of juvenile fish was higher than that of adult fish. The CJ group mainly gut bacterial community were *Proteobacteria* (34.03%), *Firmicutes* (20.13%), *Actinobacteria* (15.96%), *Bacteroidetes* (11.18%); the CA group were *Proteobacteria* (35.71%), *Fusobacteria* (34.51%), *Firmicutes* (28.91%); XJ group were *Proteobacteria* (65.97%) and *Actinobacteria* (17.43%); XA group were *Proteobacteria* (89.36%) (Fig. 2 C, Table S3). *Proteobacteria* was an absolute dominant bacteria phylum in the gut of bighead carp, whether in Chagan lake or Xinlicheng reservoir, as well as in adult or juvenile fish. *Actinobacteria* was one of the dominant bacteria in the intestinal tract of juvenile fish in both two habitats. Meanwhile, *Firmicutes* dominated in the gut of bighead carp living in Chagan lake, compared with fish living in Xinlicheng reservoir.

Genus level analysis revealed that the most dominant genera in bighead carp from Chagan lake were *Cetobacterium*, *Paraclostridium*, *Bacillus*, and the most dominant genera in bighead carp from Xinlicheng reservoir were *Acinetobacter*, *Vibrio*, *Neorickettsia* (Fig. 2 B). The gut bacterial community of bighead carp was significantly different in two habitats(*P* < 0.05). Meanwhile, the intestinal dominant genera of bighead carp were significantly different in different ages (*P* < 0.05). Four experimental groups were compared, the dominant genera of CA group were Cetobacteriu*m*, *Paraclostridium*, *Clostridium_sensu_stricto_1* and *Aeromonas*; CJ group were *Bacillus*, *Lactobacillus*, *hgcI_clade* and *Sphingomonas*; XA group were *Vibrio* and *Neorickettsia*; XJ group were *Acinetobacter*, *Cetobacterium*, *Stenotrophomonas*, *Microbacterium*, *Corynebacterium_1* and *Brevibacterium* (Fig. 2 D).

### Relationship between gut bacterial community and free amino acids or fatty acids variables in the bighead carp

**Fig. 3:**
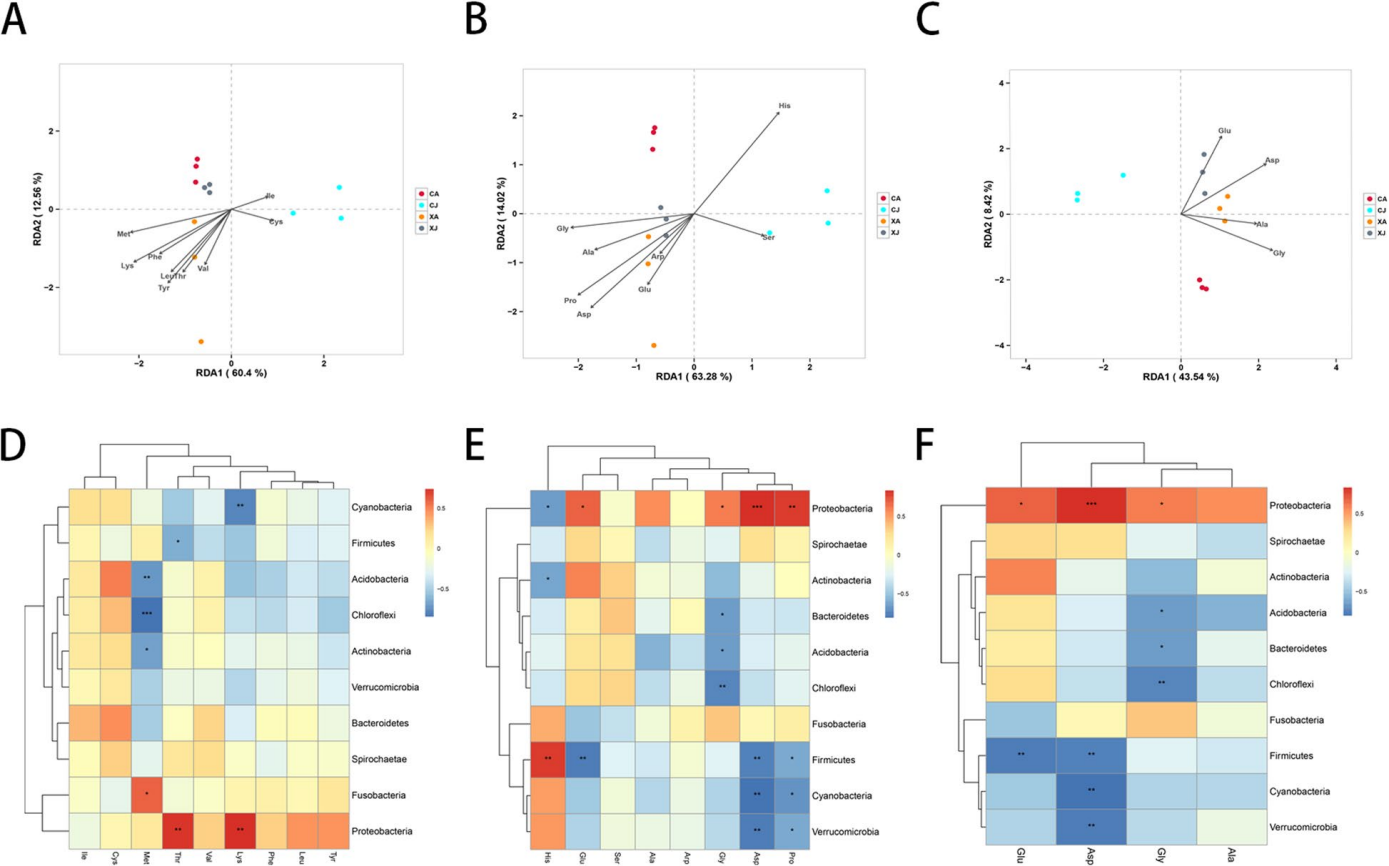
Relationship between gut bacterial community and free amino acid variables (**A**-**C**) RDA analysis different samples in relation to the free amino acids variables. (**D**-**F**) Heat-map of interrelationship between phylum level microbial species(top 10 dominant microbiota) and amino acids. (**A**, **D**) Essential amino acid. (**B**, **E**) Non-essential amino acid. (**C**, **F**) Umami amino acid. *** *P* < 0.001;** *P* < 0.01;* *P* < 0.05

**Fig. 4:**
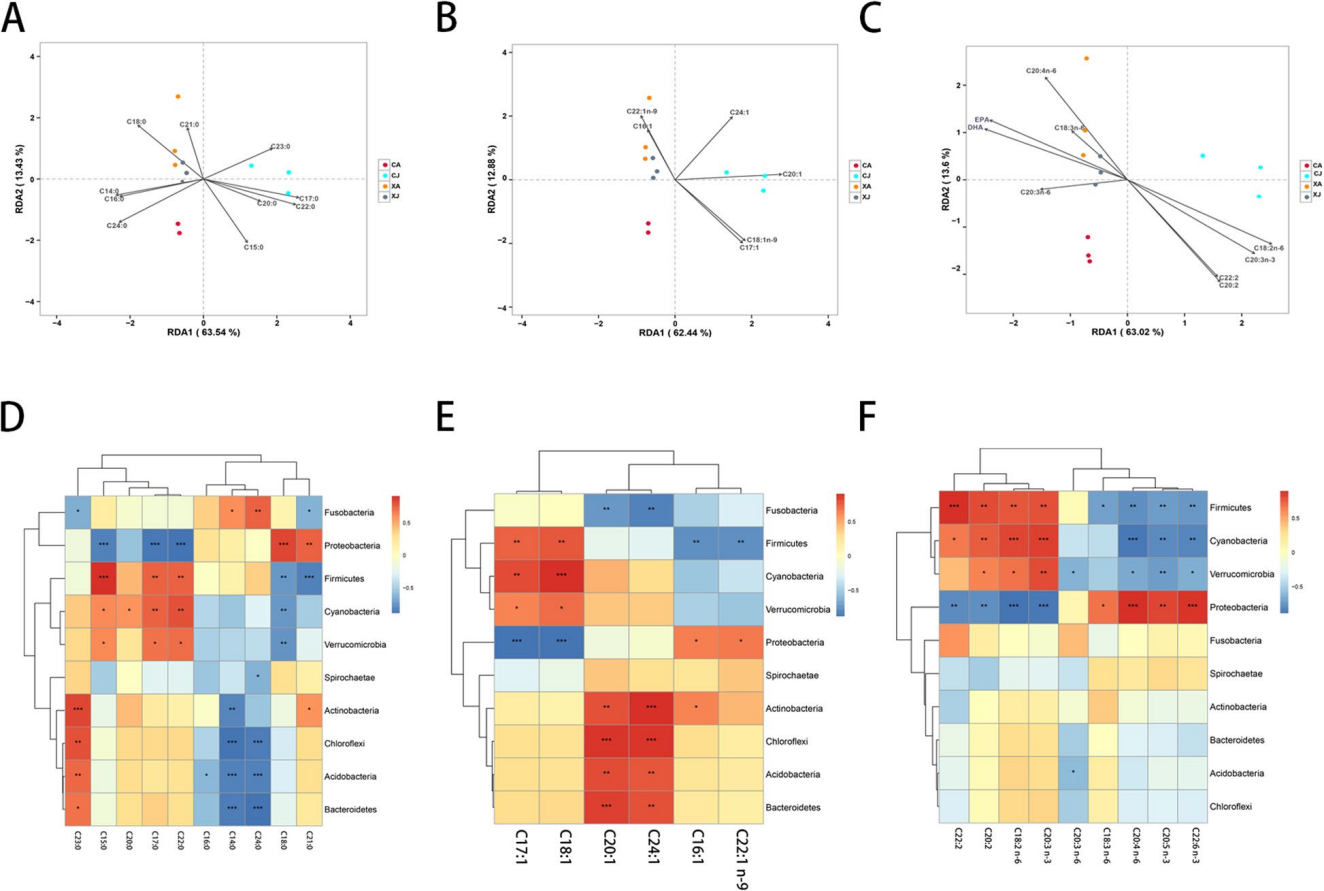
Relationship between gut bacterial community and fatty acids variables (**A**-**C**) RDA analysis different samples in relation to the fatty acids variables. (**D**-**F**) Heat-map of interrelationship between phylum level microbial species(top 10 dominant microbiota) and fatty acids. (**A**, **D**) SFAs.(**B**, **E**) MUFAs. (**C**, **F**) PUFAs. *** *P* < 0.001;** *P* < 0.01;* *P* < 0.05

To explore the main relationship for the influence of the abundance of gut bacteria in the bighead carp on the free amino acids and fatty acids in the muscle, redundancy analysis (RDA) and heat-map were used to analyze the relationship between gut bacteria community at the phylum level and the muscle free amino acids (Fig. 3) and fatty acids (Fig. 4). The relative content of the free amino acids or fatty acids in a sample could be judged by the distance between the free amino acids (Fig. 3 A-C) or fatty acids (Fig. 4 A-C) and the position of each sample. A closer distance indicated a higher relative content. In addition, the relationship between the dominant microbiota species in the top ten at phylum level and muscle parameters of free amino acids (Fig. 3 D-F) or fatty acids (Fig. 4 D-F) were analyzed using a heat-map. Red indicated that muscle parameters of free amino acids or fatty acids were positively correlated with microbial species, while blue indicated a negative correlation. The depth of the color indicates the degree of correlation. The results showed that *Proteobacteria* was positively correlated with the contents of Thr, Lys, Pro, Asp, Gly and Glu, *Actinobacteria* was negatively correlated with Met and His. EPA and DHA were positively correlated with *Proteobacteria*, EPA and DHA were not significantly associated with *Actinobacteria*.

## Discussion

Bighead carp (*A. nobilis*) belongs to the family of *Cyprinidae*, as a good source of protein for human, it is already the most important species for aquaculture and widely cultivated in China, especially in Jilin Province. Chagan lake is a typical saline-alkaline lake, bighead carp is the main species of fish. Xinlicheng reservoir is the source of drinking water for the people of Changchun city, the water quality is normal and neutral and bighead carp is abundant in it. Recently, gut microbes have become a research focus, gut microorganisms can form a relatively stable gut ecological environment in the host intestine, proliferate through nutrients provided by the host. Some bacterial metabolites can be absorbed and utilized by the host. For instance, *Bacteroidetes* play an important role in the fish intestine, as they can ferment plant-derived substrates, producing high levels of short-chain fatty acids (SCFA) that supply the host with an additional source of energy [29], and *Cetobacterium* can produce vitamin B12 and can ferment peptides and carbohydrates [30]. Bacteria maintain a relatively balanced state and the immune response of the host. It is worth noting that gut microbes vary according to genetics, geography, age, diet, and other environmental factors. Gut microbiota abundance of juvenile fish in Chagan lake(CJ group) was significantly higher than that of other groups. The gut microbial analysis demonstrated that the dominant bacteria of the bighead carp belonged to five phyla, *Proteobacteria*, *Firmicutes*, *Fusobacteria*, *Actinobacteria*, and *Bacteroidetes*. Compared with previous studies, gut microbial structure of bighead carp was similar to those of *Siniperca chuatsi*, *Silurus meridionalis* [31], *Pelteobagrus fulvidraco* [32]. But significantly different from that of mammals(*Bos taurus* [33] and human [34], etc), this result may be due to bighead carp belonging to the *chordata* phylum. *Proteobacteria* are widely distributed in the environment, *Aeromonas* belongs to *Proteobacteria*, and most members of *Aeromonas* are potential pathogens in aquaculture, like *Aeromonas veronii* [35], *Aeromonas hydrophila* [36], When the conditions are appropriate, the bacteria can cause fish disease. This result is consistent with an earlier study suggesting that gut may be a reservoir for many conditional pathogens [37]. *Firmicutes* includes bacillus (such as *bacillus subtilis* that is commonly used in aquaculture to prevent enteritis in aquatic animals and to purify water quality) and *Lactobacillus* (such as *lactobacillus casei* that is often used in the production of fermented dairy products) and else. Based on the advantages of employing *Firmicutes* in aquaculture, we speculated that the higher abundance of *Firmicutes* in the intestinal tract of bighead carp living in saline water might be a result of environmental pressure that is conducive to food ingestion and digestion in saline water. While, bacterial abundance in the intestinal tract of juvenile fish was higher than that of adult fish, probably because the immune system of juvenile fish was not sound, which led to the external and maternal effect bacterial colonies were easy to colonize. In addition, whether in Chagan lake or Xinlicheng reservoir, *Actinobacteria* was one of the dominant bacteria in the intestinal tract of juvenile fish. *Actinobacteria* are mainly distributed in the bottom mud in the water; bighead carp are filter feeders living in the middle and upper water layers, but the juvenile fish are omnivorous and live in the lower water layers. Thus it is not difficult to explain why juvenile fish intestinal tract *Actinobacteria* abundance was higher, as bighead carp experience a change in feeding habits from juvenile to adult. At the genus level, due to the difference of habitat and age, there were significant differences in intestinal microorganisms of bighead fish, and the dominant bacteria in each group were different. The specific reasons for the differences need further study and demonstration. Previous studies showed that DO%, rather than salinity, was a main environmental factor influencing microbial community diversity in a salt-freshwater transition zone [26]. In addition, a comprehensive census of lake microbial diversity on a global scale showed that alpha diversity indices of microbial community did not show significant correlations with latitude, salinity and pH [38]. In this study, the gut microbial structure of bighead carp in two lakes was similar, but the microbial content was significantly different.

It is reported that changing protein levels in food or increasing the content of free amino acids in muscle that influences the flavor of meat [39, 40]. Bighead carp is favored by consumers for its high content of unsaturated fatty acids (such as DHA and EPA) and umami taste, while the contents of different free amino acids in muscles directly affect the taste of fish. Therefore, in our study, the contents of free amino acids and fatty acids in muscle were detected. Compared with other studies on fish and mollusks, taurine was the most abundant free amino acids and follow Glu, Gly and His. Taurine has been reported to have no important effect on the formation of aroma active components [4, 41]; thus, taurine content was not detected in this study. Previously reported that free amino acids of Asp and Glu are responsible for the umami taste, and Phe for the sour taste, a sweet taste is mainly associated with amino acids such as Ala, Gly, Thr, Ser, Lys and Pro, while His, Met, Leu, Val, Arg, Ile impart a bitter taste [42–45]. The result was shown that Glu content of adult fish was lower than that of the juvenile, while His Met Arg, Phe and Leu were higher than that of juvenile fish (parts of amino acid have no significant difference). This may scientifically answer consumers’ questions about why juvenile fish tasted better. EPA and DHA are fatty acids that cannot be synthesized by human self and need to be absorbed from the outside. Our results showed that the percentage of EPA and DHA in adult fish is higher than that in juvenile fish.

Gut flora changes cause metabolic disorders that are closely related to obesity, diabetes, liver cancer [46, 47] and other diseases. Meanwhile, gut microorganisms disorders can lead to a variety of immune abnormalities and diseases [48], such as diarrhea, inflammatory bowel disease, colon cancer, rheumatoid arthritis, cardiovascular disease, etc. [49]. Moreover, microorganisms can affect some mental illnesses [50], such as autism spectrum disorders [51]. Nevertheless, whether the quality of livestock products and fishery products can be changed by intestinal microbes has not been reported. In our study, the relationship between free amino acids or fatty acids and gut microorganisms was analyzed for the first time. The results showed that *Proteobacteria* was positively correlated with Thr, Lys, Pro, Asp, Gly and Glu content, *Actinobacteria* was negatively correlated with Met and His. EPA and DHA were positively correlated with *Proteobacteria*, EPA and DHA were not significantly associated with *Actinobacteria*. Taken together, amino acids and fatty acids can influence the flavor and quality of meat, while these data suggest that intestinal microbiota may affect the content of free amino acids or fatty acids. Therefore, it is speculated that intestinal microbiota may indirectly affect the flavor and quality of meat, but more data are needed to support. This study analyzed the relationship between gut microbes and free amino acids and fatty acids at the phylum level. More research is needed to determine exactly which bacteria are responsible, what proportions of the bacteria are involved, and how they colonize in the gut.

## Conclusions

Overall, it was speculated that the contents of free amino acids and fatty acids in muscle might be affected by the difference of gut microbiota, thus affecting the taste and nutritional quality. It will provide a new perspective for gut bacterial communities to affect amino acid or fatty acids contents in muscle, accordingly influence taste. Next, we will screen the bacteria that are beneficial to the growth and digestion of fish and that influence taste; by adding them to the feed and to study how the bacteria influence fish quality.

## Materials and methods

### Water quality physicochemical analysis

Dissolved oxygen (DO), pH were determined in situ using the Hach HQ30d portable meter (Hach Co., Loveland, CO, USA). The concentration of total nitrogen (TN), total phosphorous (TP), nitrate (NO_3_-N), ammonia (NH_4_-N), and chemical oxygen demand (COD) was measured in the laboratory using methods by Xiong [52]. COD_Mn_ was measured using a fast digestion-spectrophotometric method [53]. Anions (Cl^−^ and SO_4_^2−^) and cations (Na^+^, K^+^, Ca^2+^, and Mg^2+^) were measured by suppressed ion chromatography (Dionex ICS1100). HCO_3_^−^ was analyzed by acid-based titration analysis (DZ/T 0064.49–1993). Nine repetitions were performed for each physicochemical parameter.

### Animals and sample collection

Bighead carp (*A. nobilis*) from two different lakes in Jilin Province (Chagan lake and Xinlicheng reservoir) were studied. The experimental subjects were divided into four groups: CJ (Chagan lake juvenile fish group), CA (Chagan lake adult fish group), XJ (Xinlicheng reservoir juvenile fish group) and XA(Xinlicheng reservoir adult fish group). In each group, three replicates were performed, with three fish tissue mixtures as one repetition and 18 fish from each lake (nine juvenile fish and nine adult fish). Juvenile fish with an average weight of 150 ± 25 g and adult fish with an average weight of 4500 ± 200 g were procured from two lakes. The juvenile fish were euthanized using 300 mg/L of Methane-Sulfonate − 222 (MS-222), and the adult fish were euthanized using 500 mg/L of MS-222. Tissue samples from muscle and gut were collected. The muscle was sampled for the determination of free amino acids and fatty acids, and gut was sampled for high-throughput sequencing analysis (*n* = 3, mixed three fish tissue as one sequencing sample). All of the samples were frozen in liquid nitrogen prior to storage in a − 80 °C freezer. All of the experiments and handling of the animals were conducted according to the research protocols approved by the Institutional Animal Care and Use Committee, Jilin Agricultural University.

### Determination of free amino acids

Analysis of amino acids were performed by HPLC with pre-column derivatization using phenylisothiocyanate (PITC) according to the method described by Fuentes [41]. All of the analyses described above were performed in triplicate.

### Determination of fatty acids analysis

Fatty acids were determined by gas chromatography-mass spectrometry (GC-MS). The extraction of the total lipid was carried out using a chloroform-methanol (2:1, v/v) solvent system with 0.05% BHT. Transmethylation was performed using methanol-hydrochloric acid-dimethoxypropane (40:4:1.6, v/v/v). The gas liquid chromatography system was a gas chromatograph (ThermoFisher Trace 1310 ISQ) equipped with a capillary column (TG-5MS, 30 m × 0.25 mm × 0.25 μm). Two microlitres of each extract was injected into a capillary column. Helium gas (ultrahigh purity grade, 99.999%) at a constant flow rate of 1.2 mL/min was used as the carrier gas. The injector temperature was 290 °C; the oven temperature was started at 80 °C and held for 1 min; the oven temperature was increased from 80 to 200 °C at a rate of 10 °C/min, increased to 250 °C at 5 °C/min, and finally increased to 270 °C at 2 °C /min and held for 3 min. The ion source temperature was set at 280 °C, and the ionization voltage was 70 eV; the scan range was 30–400 amu. Fatty acids in the fish muscle samples were identified by comparison of retention times and mass spectra of unknowns with standard compounds run under the same conditions. Fatty acid standard solutions were prepared following the same procedure described for the samples. Peak areas of FAMEs standards were used to quantify utilizing an external calibration curve.

### DNA extraction, PCR amplification, and high-throughput sequencing

Total bacterial DNA was extracted from samples using QIAamp DNA stool Mini Kit from Qiagen (Germany) according to the manufacturer’s protocol. The gut samples included all contents and mucus. The quality and quantity of DNA were assessed by the ratios of 260 nm/280 nm and 260 nm/230 nm, respectively. Eligible DNA was stored at − 80 °C until further treatment. The 16S rDNA gene library preparation was performed using the polymerase chain reaction (PCR) amplification of the V3–V4 region. The common primers (338F:5′-ACTCCTACGGGAGGCAGCA-3′, 806R: 5′-GGACTACHVGGGTWTCTAAT-3′) were combined with adapter sequences and barcode sequences. The thermal cycle conditions were as follows: 95 °C for 5 min (1 cycle), 95 °C for 30 s/50 °C for 30 s/72 °C for 40 s (25 cycles), and a final extension at 72 °C for 7 min. The PCR products were performed on an Illumina MiSeq platform for high-throughput pyrosequencing at Biomarker Technologies Co, Ltd. (China).

### Data analysis

High-throughput sequencing raw data were converted into sequenced reads by base calling analysis. The overlapping regions between the paired-end reads were merged using FLASH v1.2.7. Raw reads were quality filtered using Trimmomatic v 0.33 and chimera sequences were removed using UCHIME v4.2 to obtain effective tags. Based on a 97% sequence similarity, effective reads from each sample were grouped into the same operational taxonomic units (OTUs). Venn diagrams were used to show the numbers of common and unique OTUs between samples. Chao1, ACE, Simpson, and Shannon indices were calculated to compute alpha diversity. Beta diversity was calculated by employing PCA analysis on the microbial community structure. The statistics of microbial community structure at the Phylum, Class, Order, Family, Genus and Species levels of each group were carried out via QIIME. Phylogenetic diversity analysis incorporated branch lengths of taxa from a phylogenetic tree. Heat-map analysis was conducted using the R package gplots. In order to reveal patterns between bighead carp gut microbial communities and muscle free amino acids or fatty acids, redundancy analysis(RDA) was performed to compare the 10 most dominant taxa with the relevant variables. The contents of free amino acids and the percentage of fatty acids in the muscle of bighead carp were expressed as mean ± standard deviation (S.D), and differences with *P* < 0.05 were considered to be statistically significant. T-test and one-way ANOVA was performed on statistical analyses using the SPSS19.0 package.

## Supplementary Information

**Additional file 1.**

**Additional file 2.**

**Additional file 3.**

## Data Availability

All data generated or analyzed during this study are included in this published article. All raw sequences have been uploaded to NCBI under Bioproject PRJNA733887. https://dataview.ncbi.nlm.nih.gov/object/PRJNA733887
