# Supplementary material for: Comparison of gut microbial communities, free amino acids or fatty acids contents in the muscle of wild Aristichthys nobilis from Xinlicheng reservoir and Chagan lake
Source: BMC Microbiol. 2022 Jan 20;22:32. doi: 10.1186/s12866-022-02440-1 (PMC8772204; doi:10.1186/s12866-022-02440-1)
Supplement: Supplementary file 1 — Additional file 1. [file 12866_2022_2440_MOESM1_ESM.docx]

| Sample ID | PE Reads | Raw Tags | Clean Tags | Effective Tags | AvgLen(bp) | GC(%) | Q20(%) | Q30(%) | Effective(%) |
| --- | --- | --- | --- | --- | --- | --- | --- | --- | --- |
| CA1 | 79967 | 75971 | 70130 | 69221 | 414 | 51.58 | 96.94 | 94.2 | 86.56 |
| CA2 | 80143 | 76299 | 70808 | 70333 | 417 | 52.43 | 97.03 | 94.35 | 87.76 |
| CA3 | 79969 | 76151 | 70558 | 69781 | 414 | 52.12 | 96.97 | 94.28 | 87.26 |
| CJ1 | 53831 | 51687 | 47797 | 46267 | 417 | 54.71 | 96.95 | 94.2 | 85.95 |
| CJ2 | 62075 | 59369 | 54742 | 52534 | 419 | 54.29 | 96.92 | 94.15 | 84.63 |
| CJ3 | 58750 | 56065 | 51478 | 49610 | 419 | 54.18 | 96.83 | 94.01 | 84.44 |
| XA1 | 79753 | 76310 | 68373 | 68174 | 429 | 52.11 | 96.44 | 93.35 | 85.48 |
| XA2 | 79914 | 75489 | 66525 | 66292 | 424 | 54.58 | 96.16 | 92.77 | 82.95 |
| XA3 | 65952 | 61810 | 54360 | 54166 | 418 | 53.72 | 95.89 | 92.19 | 82.13 |
| XJ1 | 74843 | 70115 | 61285 | 60951 | 423 | 53.04 | 96.07 | 92.55 | 81.44 |
| XJ2 | 79940 | 76216 | 67912 | 67451 | 424 | 53.38 | 96.42 | 93.18 | 84.38 |
| XJ3 | 66137 | 62311 | 55104 | 54754 | 421 | 52.69 | 96.31 | 92.98 | 82.79 |

Table S1：Quality assessment of sequencing data
