# Supplementary material for: Comparison of gut microbial communities, free amino acids or fatty acids contents in the muscle of wild Aristichthys nobilis from Xinlicheng reservoir and Chagan lake
Source: BMC Microbiol. 2022 Jan 20;22:32. doi: 10.1186/s12866-022-02440-1 (PMC8772204; doi:10.1186/s12866-022-02440-1)
Supplement: Supplementary file 2 — Additional file 2. [file 12866_2022_2440_MOESM2_ESM.docx]

Table S2: Abundance of gut bacterial community in phylum level

| phylum | Chagan Lake | Xilicheng Reservoir |
| --- | --- | --- |
| Proteobacteria | 35.18% | 77.89% |
| Firmicutes | 26.13% | 4.12% |
| Fusobacteria | 23.97% | 4.91% |
| Actinobacteria | 5.07% | 9.26% |
| Bacteroidetes | 3.73% | 1.79% |
| Cyanobacteria | 2.25% | 0.08% |
| Spirochaetae | 0.23% | 1.20% |
| Acidobacteria | 1.20% | 0.03% |
| Verrucomicrobia | 0.55% | 0.03% |
| Chloroflexi | 0.37% | 0.02% |
| Others | 1.32% | 0.68% |
