# Supplementary material for: Comparison of gut microbial communities, free amino acids or fatty acids contents in the muscle of wild Aristichthys nobilis from Xinlicheng reservoir and Chagan lake
Source: BMC Microbiol. 2022 Jan 20;22:32. doi: 10.1186/s12866-022-02440-1 (PMC8772204; doi:10.1186/s12866-022-02440-1)
Supplement: Supplementary file 3 — Additional file 3. [file 12866_2022_2440_MOESM3_ESM.docx]

Table S3: Abundance of gut bacterial community in phylum level

| phylum | CA | CJ | XA | XJ |
| --- | --- | --- | --- | --- |
| Proteobacteria | 35.71% | 34.02% | 89.35% | 65.97% |
| Firmicutes | 28.91% | 20.15% | 2.16% | 6.16% |
| Fusobacteria | 34.51% | 1.30% | 1.96% | 7.97% |
| Actinobacteria | 0.02% | 15.97% | 1.39% | 17.42% |
| Bacteroidetes | 0.26% | 11.19% | 2.17% | 1.39% |
| Cyanobacteria | 0.28% | 6.48% | 0.04% | 0.12% |
| Acidobacteria | 0.00% | 3.81% | 0.02% | 0.04% |
| Spirochaetae | 0.01% | 0.72% | 2.26% | 0.10% |
| Verrucomicrobia | 0.23% | 1.25% | 0.03% | 0.02% |
| Chloroflexi | 0.00% | 1.12% | 0.01% | 0.03% |
| Others | 0.08% | 4.01% | 0.60% | 0.77% |
